# Efficacy and Safety of Levetiracetam and Carbamazepine as Monotherapy in Partial Seizures

**DOI:** 10.1155/2015/415082

**Published:** 2015-12-20

**Authors:** Swaroop Hassan Suresh, Ananya Chakraborty, Akash Virupakshaiah, Nithin Kumar

**Affiliations:** ^1^Cipla Ltd, 117/1, Anjanadri, Pantharapalya, Bangalore 560039, India; ^2^Department of Pharmacology, Vydehi Institute of Medical Sciences and Research Centre, No. 82 EPIP Area, Whitefield, Bangalore 560037, India; ^3^Department of Neurology, Columbia Asia Hospital, Whitefield, Bangalore 560066, India

## Abstract

*Introduction*. Levetiracetam (LEV) is a newer antiepileptic drug with better pharmacokinetic profile. Currently, it is frequently used for the treatment of partial seizures. The present study was undertaken to compare the efficacy and safety of LEV and Carbamazepine (CBZ) in partial epilepsy.* Methods*. This was a prospective, open labeled, randomized study. It was conducted in participants suffering from partial seizures after the approval of ethics committee and written informed consent. The first group received Tab LEV (500 to 3000 mg/day) and the second group received Tab CBZ (300 to 600 mg/day). The primary outcomes were efficacy and safety. The secondary outcome was the Quality of Life (QOL). Efficacy was assessed by comparing the seizure freedom rates at the end of 6 months. Safety profile was evaluated by comparing the adverse effects. QOL was assessed by QOLIE-10 scale.* Results*. The overall seizure freedom rate at the end of 6 months was 71.42% in CBZ group compared to 78.57% in LEV group (*p* = 0.2529). Both LEV and CBZ reported a similar incidence of adverse reactions. LEV group reported more behavioral changes like increased aggression and anxiety. Also, it showed better QOL compared to the CBZ group.* Conclusion*. LEV monotherapy and CBZ monotherapy demonstrated similar efficacy for treatment of partial epilepsy and were found to be well tolerated.

## 1. Introduction

Epilepsy is a chronic disorder characterized by 2 or 3 recurrent seizures of cerebral origin. It is the second most common neurological condition after headache. The estimated average prevalence of epilepsy is 6.8 per 1000 people in US, 5.5 per 1000 people in Europe, and 1.5 to 14 per 1000 people in Asia, respectively. Epilepsy is classified based on the source of seizure into partial and generalized seizures [1]. World Health Organization (WHO) and International League against Epilepsy (ILAE) have estimated that, out of 50 million people, 34 million with epilepsy live in developing countries. Out of them, nearly 80% are not on treatment [2]. In India, it is estimated that, out of over 1.23 billion population, there are around 6–10 million people with epilepsy. It accounts for nearly 1/5th of global epilepsy burden [3]. Epilepsy is classified based on the source of seizure into partial and generalized seizures. Partial seizures arise in specific, often small, loci of cortex in one hemisphere of the brain. About 2/3rd of newly diagnosed epilepsies are partial or secondarily generalized. The treatment of the epilepsy depends on appropriate classification of seizure type and the epileptic syndrome [4].

The mainstay of treatment of epilepsy is pharmacological therapy with antiepileptic drugs (AEDs). In epilepsy, optimal treatment is important as the condition is associated with increased morbidity and mortality and unexpected deaths without clear structural or pathological cause [5, 6]. AEDs are selected based on the nature of the disease, the efficacy and tolerability of the agent, and the characteristics of the patient [7]. Treatment options for epilepsy include the older AEDs (carbamazepine, ethosuximide, phenytoin, phenobarbital, primidone, and valproic acid) as well as several newer drugs (Levetiracetam, felbamate, gabapentin, lacosamide, lamotrigine, oxcarbazepine, pregabalin, rufinamide, tiagabine, topiramate, vigabatrin, and zonisamide) [8]. Carbamazepine (CBZ) is the preferred drug for the treatment of partial seizures but it has the disadvantages of requirement for frequent dosing, dose related adverse reactions, and drug interactions. Recently, Levetiracetam (LEV) has become one of the most frequently prescribed newer drugs for the treatment of partial seizures. It offers several advantages like twice daily dosing, better safety profile, less drug interactions, and no requirement of serum level monitoring. This advantageous pharmacologic profile makes LEV an attractive first-line or adjunctive therapy for epileptic seizures [9, 10].

Till date, there have been a very few studies on the efficacy and safety of LEV and CBZ in partial epilepsy. Hence, this study was undertaken to compare the efficacy and safety of LEV and CBZ as monotherapy in partial epilepsy.

## 2. Materials and Methods

### 2.1. Study Design and Setting

This was a randomized, prospective, open label, comparative monotherapy study. The study was conducted in the Department of Neurology at Vydehi Institute of Medical Sciences and Research Center, Bengaluru, India. The institute is a 1000-bed tertiary care hospital equipped with modern diagnostic and treatment facilities. Patients visiting this hospital come from different geographical regions including Southern Karnataka, Andhra Pradesh, and West Bengal, India, with a fair representation of both urban and rural populations. The patients belong to varied socioeconomic strata. The study was conducted after receiving the approval from the Institutional Ethics Review Board. The duration of the study was one year from January 2013 to December 2013.

### 2.2. Selection of the Participants

The participants were included in the study after obtaining written informed consent. The study inclusion criteria included subjects of age between 18 and 60 years diagnosed newly with focal or partial seizures with or without secondary generalization. The exclusion criteria were pregnant and lactating mothers, patients with nonepileptic seizures, auras or absence of seizures, and patients with acute symptomatic seizures occurring within 14 days of an acute brain injury such as stroke and patients with history of psychiatric illness.

### 2.3. Data Collection

The Neurology OPD was used to recruit participants with newly diagnosed partial epilepsy. The study objectives and process were explained to the patients or their relatives in their own language. Subjects who consented to participate were then interviewed and were divided into two groups by the toss of a coin. Each group recruited 30 participants. Group 1 participants were prescribed Tab LEV, 1000–3000 mg/day/oral; and group 2 participants were prescribed Tab CBZ, 400–1200 mg/day/oral. The participants were started with minimum dose, 500 mg of LEV and 200 mg of CBZ, given twice daily after food and then titrated depending on the seizure control. LEV dose was increased by 500 mg twice daily every 2 weeks up to a maximum of 3000 mg/day if seizure control was not achieved. Similarly, CBZ dose was increased by 200 mg twice daily up to a maximum of 1200 mg/day if seizure control was not achieved. In cases where the seizure was not controlled after titration of drug dose, the participant was shifted to adjuvant therapy based on the clinical condition. The participant was also discontinued from the study.

All the participants were given a diary and were asked to note down any adverse effects (AE). They were advised to come after 4, 12, and 26 weeks after the initiation of therapy for follow-up. During follow-up visits, the participants were thoroughly examined, history of breakthrough seizures was elicited, and any AEs were noted. QOL was assessed by using the QOLIE-10 questionnaire before initiation of the treatment and after 26 weeks of therapy [11]. QOLIE-10 comprises seven components: (1) seizure worry, (2) overall QOL, (3) emotional well-being, (4) cognitive function, (5) energy/fatigue, (6) medication effects: physical effects and psychological effects, and (7) social functioning: work, driving, and social function. The English version of QOLIE-10 was used for this study. Participants who were conversant in English completed the questionnaire themselves. Since the remaining patient population was multilingual (Kannada, Hindi, Bengali, and Telugu), the questions were explained to them in their respective languages and responses were elicited. The responses were then scored to provide subscale scores which were then averaged to provide a total score.

### 2.4. Data Analysis

The baseline data like demography, efficacy, and AEs were subjected to descriptive statistical analysis and expressed as mean ± SD, frequencies, and percentages. The QOLIE-10 scores were expressed as mean ± SD scores. The categorical variables were compared using Chi-square (*χ* ^2^) test. Comparison of continuous variables between groups was carried out using unpaired Student's *t*-test. Statistical significance was set at *p* < 0.05.

## 3. Results

A total of 79 subjects were screened for the study. Out of them, 60 (75.6%) participants who fulfilled the eligibility criteria were randomized into the two study groups. Following is the summarization of the observed results.

### 3.1. Patient Characteristics and Demographic Profile

Out of 30 participants in CBZ group, 17 were male and 13 were female. Out of 30 participants in LEV group, 13 were male and 17 were female. The mean age of the male participants in CBZ group was 30.70 ± 2.66 years, and in the LEV group it was 22.62 ± 1.152 years (*p* value, 0.0834). The mean age of females in CBZ group was 29.31 ± 2.44 years and in LEV group it was 28.18 ± 2.553 yrs (*p* value, 0.7101). Thus there was no significant difference between the mean age of males and that of females in both groups. The mean BMI of CBZ group was 22.56 ± 0.41 kg/m^2^ and that of LEV group was 21.49 ± 0.41 kg/m^2^ (*p* value of 0.0690). There was no significant difference in BMI in both groups.

### 3.2. Treatment Efficacy

**Figure 1:**
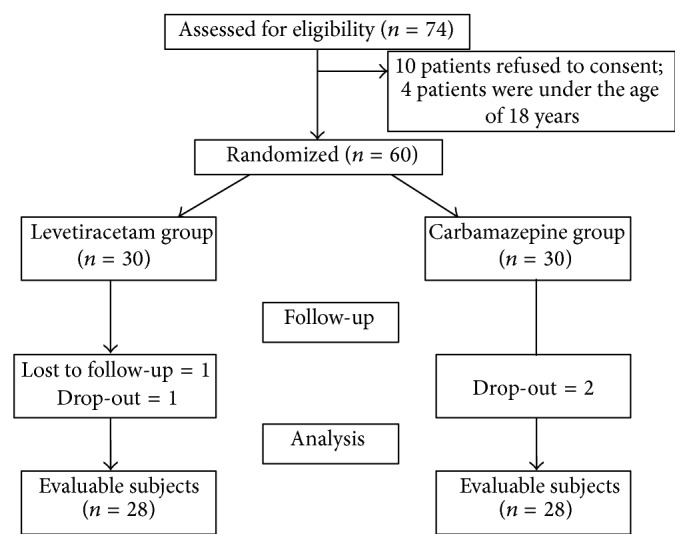
Patient disposition in the study.

Thirty participants were randomized to both CBZ group and LEV group. In the LEV group, 2 participants dropped from the study, one was lost to follow-up, and one subject had serious AE. Thus, a total of 28 subjects in LEV group were assessed for efficacy. Similarly, 2 subjects in CBZ group were dropped from the study due to AE. Thus, a total of 28 subjects from CBZ group were assessed for efficacy as shown in Figure 1.

**Figure 2:**
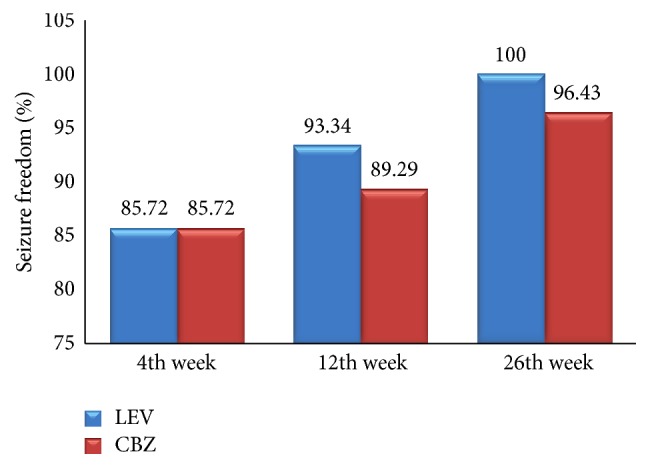
Seizure freedom at 4th, 12th, and 26th weeks.

**Figure 3:**
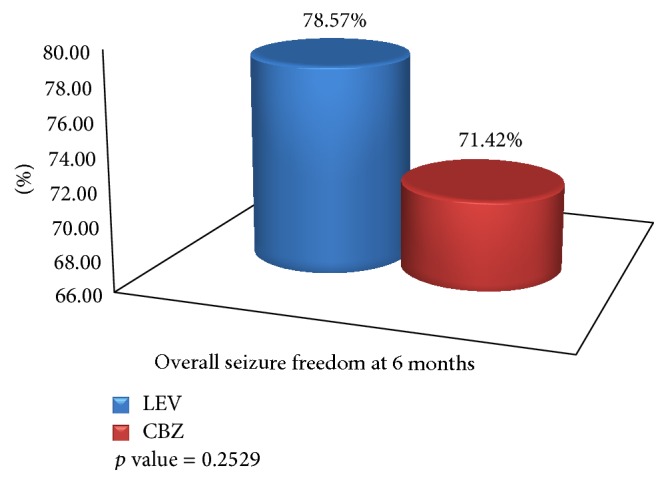
Overall seizure freedom at the end of 6 months.

All participants were followed up at 4, 12, and 26 weeks after the initiation of monotherapy. At the 4th week of follow-up, both groups had equal seizure freedom of 85.72% which is not statistically significant (*p* value of 1.000). At 12 weeks of follow-up, CBZ group had 89.29% of seizure freedom compared to LEV group which had 93.34% seizure freedom which is not statistically significant (*p* value, 0.4595). Twenty-two (78.57%) of those taking LEV and 20 (71.42%) subjects on CBZ were seizure-free for at least 6 months during the monotherapy treatment, which is not statistically significant (*p* value, 0.2529). The data is shown in Figures 2 and 3.

### 3.3. Treatment Safety

Participants who experienced at least one AE constituted 36.66% in CBZ group and 40% in the LEV group (*p* value, 0.7714), which is not statistically significant. One participant (3.33%) on LEV therapy discontinued the treatment due to AE of increased nausea and vomiting and 2 patients (6.66%) discontinued the treatment due to AE of dizziness and increased nausea. In LEV group, 5 participants experienced behavioral changes like increased aggressive behavior, 1 participant experienced suicidal tendency, 3 participants had increased anxiety, 3 participants suffered from increased sleep, 2 participants reported weight gain of around 3–5 kilograms in 3 months of duration, and 2 participants reported constipation. The other AEs reported were giddiness, decreased sleep, nausea, itching, and vomiting. In CBZ group, 6 participants experienced somnolence, and 4 patients reported dizziness. The other adverse events reported were constipation, itching, poor concentration, nausea, and vomiting.

### 3.4. QOL Assessment

In clinical practice, QOLIE-10 score ranges from 0 to 100. A total score range of less than 50 indicates the poor quality of life, a score from 50 to 70 indicates the optimal QOL, and a score more than 70 implies better QOL. QOL assessment was done in the participants in both groups at 0 weeks and at the end of 24 weeks. The mean QOL score in CBZ group at 0 weeks was 31.14 ± 1.83 and in the LEV group it was 29.76 ± 1.71 (*p* value, 0.5861) which is not statistically significant. The mean QOL score in CBZ group at the end of 26th week was 58.41 ± 1.89 and the mean QOL score in LEV group at the end of 26th week was 64.58 ± 2.02 (*p* value of 0.0302, *p* < 0.05) which was found to be statistically significant.

Overall characteristics are shown in Table 1.

## 4. Discussion

The aim of AED treatment is to achieve seizure freedom with minimal or ideally no AE and with an optimal QOL. Numerous AEDs are licensed as monotherapy for focal seizure in adults. These include the older AED like CBZ. Even though CBZ has many AEs and tolerability issues, it was considered as gold standard first-line drug to treat focal seizures from past many years. In 2013, ILEA has produced an updated review in epilepsy treatment, which highlighted the fact that newer AEDs like LEV and zonisamide have class 1-2 evidence to be used as monotherapy. This is based on regulatory trials showing noninferiority when compared to CBZ for 6-month remission [2, 12]. Till date, there have been very few studies to compare the efficacy and tolerability of LEV versus CBZ as monotherapy in focal seizures all over the world. Since the usage of LEV is very high in India, comparative study of efficacy and tolerability of LEV versus CBZ was expected to give more confidence for the use of the drug. Evaluation of QOL outcomes has been increasingly adopted into the standard management plan for epilepsy along with traditional measures of seizure frequency and AE.

### 4.1. Demographic Profile

The study sample was characterized by its relatively younger age (mean age, 27 ± 2.62 years). The mean age group of males in CBZ group was 30.70 ± 2.66 years and that of LEV group was 22.62 ± 1.152 years. The mean age group of females in CBZ group was 29.31 ± 2.44 years and that of LEV group was 28.18 ± 2.553 years. This is different from the previous studies conducted at developed countries like UK, USA, and Germany, which had relatively older age that varied from 35 to 40 years, respectively. In this study, the CBZ group comprised 17 (56.66%) males and 13 (43.33%) females and LEV group comprised 13 (43.33%) males and 17 (56.66%) females, respectively. This is comparatively similar to the study conducted by Brodie et al., which had 58.8% males and 41.2% females in CBZ group and 51.2% males and 48.8% females in LEV group [9].

### 4.2. Efficacy Outcome

In this study, the efficacy was mainly assessed by seizure freedom rate. According to ILAE, a patient is considered as seizure-free following an intervention after a period without seizures has elapsed equal to three times the longest preintervention interseizure interval over the previous year [12]. In this study, we assessed seizure freedom rate at 4, 12, and 26 weeks. We also assessed the overall seizure freedom rate at the end of 6 months of the study; similarly, in LaLiMo trial, they have assessed seizure freedom rates at 6, 16, and 26 weeks [12].

Participants were asked to come for follow-up visits at 4, 12, and 26 weeks after initiation of the drug. The pretreatment mean seizure rate in LEV was 4.2 ± 0.65 per month which was comparatively higher than CBZ group with 2.83 ± 0.19 per month. At 4 weeks of follow-up, the seizure freedom rate in both CBZ and LEV groups was the same (85.72%). Since the pretreatment seizure frequency in LEV group was high, the seizure freedom at 4 weeks goes in the favor of LEV group. Similarly, in LaLiMo trial, the seizure freedom at 6 weeks in LEV group was 83.6% compared to 79.8% in Lamotrigine group (*p* = 0.47) with no statistical significance [12]. In this study, both groups showed better seizure freedom even though the results were not statistically significant. The increased seizure freedom may be due to better drug adherence.

Seizure freedom at 12 weeks of therapy in LEV group was 93.34%, while in CBZ group it was 89.29% (no statistical significance; *p* = 0.4595). Similarly, in LaLiMo trail, the seizure freedom at 16 weeks of maintenance therapy was 51.9% in LEV group and 55.7% in Lamotrigine group. Also, there was breakthrough seizures between 6 weeks and 16 weeks. Therefore, the seizure freedom rates in both groups had reduced significantly; this might be due to lack of drug adherence [12]. In this study, the seizure freedom at 12 weeks was comparatively better in LEV group than in CBZ group, even though the results were not statistically significant.

In most of the comparative studies of LEV versus CBZ, the main efficacy outcome was seizure freedom rate at 6 months and 12 months. Since this was time bound academic study, we could not follow up the cases for long term.

The final efficacy outcome was assessed on seizure freedom at the end of 6 months. In our study overall seizure freedom rate at the end of 6 months was 71.42% in CBZ group compared to 78.57% in LEV group (*p* = 0.2529), which is not statistically significant. As per Perry et al.'s study, where they have compared LEV versus CBZ as monotherapy for partial epilepsy, the efficacy outcome was seizure freedom at 6, 12, and 24 months. The seizure freedom rate at the end of 6 months was 73% in LEV group compared to 65% in CBZ group (*p* = 0.58) which showed no statistical significance like our study [10]. Similarly, in KOMET trial, the authors compared LEV with CBZ in newly diagnosed focal epilepsy; seizure freedom rate at 6 months for CBZ was 62% which was comparatively higher than LEV which had seizure freedom rate of 57.5%. The results were not statistically significant [13]. In another similar study by Brodie et al., the primary efficacy endpoint was seizure freedom rate at 6 months. At the end of 6 months, the seizure freedom rate was 73% in LEV group and 72.8% in CBZ group, which was almost similar efficacy in both groups [9].

No drug has shown superior efficacy to CBZ in randomized, head to head comparison in newly diagnosed epilepsy patients with partial or generalized tonic-clonic seizures. Though most of the studies clearly mention that newer AEDs are always comparable with older AEDs in efficacy, none of the studies till date showed a superior efficacy with newer AEDs compared to older AEDs. In our study too similar results were obtained; that is, LEV was comparable with CBZ in efficacy but it was not superior to CBZ.

### 4.3. Safety

The ultimate goal of treatment of epilepsy is the fact that patients should not have seizures, less AE, and an optimal QOL. In this study, both LEV and CBZ were well tolerated as initial monotherapy. Only 6.66% of patients on CBZ and 3.33% of patients on LEV withdrew from the study due to AE. There was more withdrawal of patients in the CBZ group which correlates to a previous study conducted by Brodie et al. In that study, 19.2% of patients on CBZ versus 14.4% of patients on LEV discontinued due to AE [9]. In this study, AEs were more reported from LEV (40%) compared to CBZ (36.66%) group even though the difference was not statistically significant. Similarly, in the KOMET trial, there was increased serious AE associated with LEV (13.7%) compared to CBZ group (8.2%) [13]. In contrast to these findings, in a study conducted by Perry et al., 70% of patients on CBZ experienced ADRs compared to 45% of those on LEV [10].

In this study, the participants taking CBZ mostly reported AEs like increased sleep (20%) and dizziness (13.33%) similar to the study conducted by Perry et al., where 40% of patients on CBZ reported increased sleep and 10% of patients reported dizziness. There was withdrawal of 2 subjects after 24 hours of initiation of drug, but none of the patients on CBZ reported serious AE.

In this study, subjects assigned to the LEV group most commonly (17.85%) reported behavioral changes in terms of increased aggressive behavior, increased anxiety, and suicidal tendency. Similarly, in Perry et al.'s study, LEV was associated with increased behavioral changes in terms of irritability (30.5%). Many of the case reports do suggest that LEV is associated with increased behavioral changes [14, 15]. Also, the package insert of LEV mentions the fact that LEV is contraindicated in patients with past history of psychiatric illness. A study on the safety profile of LEV mentions that 13.3% patients on LEV reported behavioral symptoms in terms of agitation, hostility, aggressiveness, anxiety, apathy, emotional liability, and depression [16]. Similarly, another study addressing the clinical experience of LEV also mentions that 33.33% of patients reported nervousness or irritability after the initiation of the drug. Also, 16.66% of patients discontinued the treatment due to the irritability [8].

In this study, 2 patients on LEV reported weight gain of 3–5 kg in 3 months. Till date, there have been reports of LEV induced weight loss. Here, weight gain can be correlated with improved QOL. Other AEs observed in this study were giddiness, increased sleep, itching, and nausea.

Long term AEs of CBZ have been reported to be leukopenia, hyponatremia, disturbances of vitamin D metabolism, agranulocytosis, and hepatitis. LEV is a comparatively new drug. The studies till date mention that the drug is well tolerated on long term use. There are reports of discontinuation of the drug due to irritability but this was related to previous history of mood disorders [17, 18]. In this regard, LEV appears to be a better option compared to CBZ for long term use. To avoid the behavioral AE, prescribers should thoroughly evaluate a patient of past psychiatric illness.

### 4.4. Quality of Life

The QOL evaluation is a relatively new measure to evaluate patient related outcome of treatment for epilepsy. Recently, other studies have tried to determine the effects of various demographic and clinical variables on the overall QOL among patients with epilepsy [2]. Here, we evaluated QOL with QOLIE-10 and studied the impact of both LEV and CBZ before and after the initiation of therapy. The mean score in CBZ group before the initiation of the therapy was 31.14 ± 1.83 compared to LEV group where it was 29.76 ± 1.71 (*p* = 0.5861) which is statistically not significant. The less scores correlate with the poor QOL of patients. After completing the course of therapy of 6 months, there was an increase in the mean score of both groups which was statistically significant. The mean score in CBZ group at the end of 6 months of initiation of therapy was 58.41 ± 1.89 compared to 64.58 ± 2.02 (*p* = 0.0302, *p* < 0.05), which was statistically significant. Unlike the previous KOMET trial, where QOL was assessed by QOLIE-31 scale, there were no clear differences between LEV and CBZ in the impact on health related quality of life [13]. Among both drugs, LEV has been shown to be superior to CBZ in terms of QOL, which can be due to the fact that LEV was associated with increased seizure freedom compared to CBZ. This increased seizure frequency can be correlated with decreased QOL in CBZ group. Similarly, another study conducted by Thomas et al. suggests that patients on monotherapy have a significant better QOL [2].

LEV thus demonstrated better QOL after 6 months of therapy compared to CBZ.

## 5. Conclusion

The efficacy of LEV was found to be comparable to CBZ as monotherapy in the treatment of partial seizures. LEV did not show superior efficacy compared to CBZ. Both drugs equally reduced the seizure frequency compared to pretreatment seizure frequency. LEV was equally tolerable to CBZ. LEV and CBZ demonstrated equal incidence of AE. LEV can be safely used as monotherapy in the treatment of partial epilepsy.

## Figures and Tables

**Table 1:** Overall characteristics of patients on Levetiracetam and Carbamazepine monotherapy.

	CBZ group	LEV group	*p* value
	*n* = 28	*n* = 28	
Male mean age	30.70 ± 2.66 yrs	22.62 ± 1.152 yrs	0.0834
Female mean age	29.31 ± 2.44 yrs	28.18 ± 2.553 yrs	0.7101
Mean BMI	22.56 ± 0.41	21.49 ± 0.41	0.0690
Pretreatment mean seizure frequency	2.83 ± 0.19	4.2 ± 0.65	0.0470
Seizure freedom at 4 weeks	85.72%	85.72%	1.0000
Seizure freedom at 12 weeks	89.29%	93.34%	0.4595
Seizure freedom at 26 weeks	96.43%	100%	0.1212
Overall seizure freedom at 6 months	71.42%	78.57%	0.2529
QOL at 0 weeks	31.14 ± 1.83	29.76 ± 1.71	0.5861
QOL at 26th week	58.41 ± 1.89	64.58 ± 2.02	0.0302
